# A Case of Maternal Impression

**Published:** 1887-07

**Authors:** S. W. Stiles

**Affiliations:** Georgia


### A CASE OF MATERNAL IMPRESSION.

BY S. W. STILES, M. D., GEORGIA.

During the Summer of 1885, while I was practicing medicine in the lower part of Meriwether County, in this State, whilst going my daily rounds among my patients, it became my good fortune to come in contact with one of the most singular freaks of Nature, the like of which I have never read in medical literature, in the person of a beautifully formed young lady, about twenty years of age, the daughter of Mr. M——, a farmer of that county. The young lady possesses more intelligence than is ordinarily found in persons of her station. At the early age of seven years she taught herself to read and write. She menstruated freely as soon as she reached the age of puberty, and has been perfectly regular in her catamenial discharges up to the present time. Her mother, as I gleaned the history of the case from responsible parties, became frightened, whilst this child was yet in uteruo, at the actions of a drunken man, and attempted to rush into the house but fell and swooned away on the door-sill. This occurred but two months before the birth of this child. The child, when born, had all the motions of a drunken man, which has continued to the present time without having abated one iota. When walking she reels from side to side and has to cling to different objects to steady herself, and in a sitting posture she can hardly support herself on a chair, her whole body sways to such a degree ; her head falls from side to side with a rotary motion ; yet she does all the cooking and washing for the entire family, and enjoys good health.

I would like to hear the opinion of the medical fraternity on this wonderful case.

